# Mesenteric venous thrombosis in a pregnant woman at first trimester gestation: a case report

**DOI:** 10.1093/jscr/rjac294

**Published:** 2022-06-21

**Authors:** Rodrigo Piltcher-da-Silva, Vivian Laís Sasaki, João Francisco Petry, Guilherme Vieceli Rhoden, Matheus Antonio Chiconelli Zangari, Mariana Piltcher-Recuero, Gabriela de Melo Rocha, Paulo Cesar Andriguetto, Yan Sacha Aguilera, Júlio Cezar Uili Coelho

**Affiliations:** General and Digestive Surgery Service, Hospital Nossa Senhora das Graças, Curitiba, Brazil; General and Digestive Surgery Service, Hospital Nossa Senhora das Graças, Curitiba, Brazil; General and Digestive Surgery Service, Hospital Nossa Senhora das Graças, Curitiba, Brazil; General and Digestive Surgery Service, Hospital Nossa Senhora das Graças, Curitiba, Brazil; General and Digestive Surgery Service, Hospital Nossa Senhora das Graças, Curitiba, Brazil; General and Digestive Surgery Service, Hospital Nossa Senhora das Graças, Curitiba, Brazil; Radiology Service, Hospital Nossa Senhora das Graças, Curitiba Brazil; General and Digestive Surgery Service, Hospital Nossa Senhora das Graças, Curitiba, Brazil; General and Digestive Surgery Service, Hospital Nossa Senhora das Graças, Curitiba, Brazil; General and Digestive Surgery Service, Hospital Nossa Senhora das Graças, Curitiba, Brazil

**Keywords:** pregnancy, mesenteric vein thrombosis, acute abdomen, vein thrombosis, mesenteric ischemia

## Abstract

Mesenteric vein thrombosis (MVT) in a pregnant patient is a rare condition that seems to be associated with the pregnancy pró-thrombotic state. This can lead to severe circumstances such as intestinal hemorrhagic ischemia, sepsis, abortion and death. Abdominal assessment is challenging due to the anatomical and physiological changes during pregnancy. MVT clinical and complementary evaluation are nonspecific, making essential an image exam. We report a case of a 33-years-old woman at 11 weeks of gestation. She sought medical evaluation due to abdominal pain and had an appendicitis diagnosis, which was treated by laparoscopic surgery. One week later, she came back complaining of nonspecific abdominal pain. So an extensive evaluation was made, and the diagnosis of MVT and intestinal ischemia was concluded. She underwent laparotomy exploration and anticoagulation, having a good evolution and so was discharged on the sixth post-operative day.

## INTRODUCTION

While mild abdominal pain is common, severe abdominal pain during pregnancy is usually an ominous sign that needs prompt management to avoid harm to maternal and fetal health [1, 2]. The pregnancy increases 5-fold the risk of thrombosis compared with nonpregnant women [3, 4]; this and the blood stasis secondary to vena cava compression by the gravid uterus explain the well-known relationship with deep vein thrombosis.

Nonetheless, mesenteric vein thrombosis (MVT) is a rare and not yet well understood potentially life-threatening condition in pregnant patients [2]. As a consequence of anatomical changes, abdominal assessment during the pregnancy is challenging and complementary evaluation with laboratory and imaging exams is associated with low threshold [1, 5]. The incidence of acute appendicitis is up to 0.2% in pregnant women and is the main cause of acute abdomen during pregnancy [1]. In the current literature, there are a few case reports where appendicitis is followed by MVT. In fact, the association between these two conditions is low [6–8].

We present a case of a first trimester pregnant woman diagnosed with MVT and bowel loop ischemia 1 week after laparoscopic appendectomy.

## PRESENTATION OF CASE

A 33-year-old woman, in the 11th week of pregnancy, presented with 5-day history of abdominal pain that has worsened in the last hours. She also referred anorexia and diarrhea. There were no obstetric or gynecological complaints. At evaluation, she was stable and had pain on right iliac fossa with negative Blumberg sign. She denied previous morbidities or surgeries. Her first child was born a year ago by normal delivery of an uneventful pregnancy.

Abdominal ultrasonography examination confirmed the diagnosis appendicitis. She underwent laparoscopic appendicectomy, and catarrhal appendicitis was identified. The surgery was uneventful, and the patient was discharged on the first post-operative day (POD).

Eight days later, she returned complaining of diffuse abdominal pain within 3 days of evolution. She had stable signs, pain on mesogastrium palpation, without peritoneal irritation. Laboratory exam with mild leukocytosis and increased C-reactive protein (CRP) (28 mg/dl) and abdominal ultrasonography showed small amounts of free fluid in the pelvis and parietal thickening in a small bowel loop.

**Figure 1:**
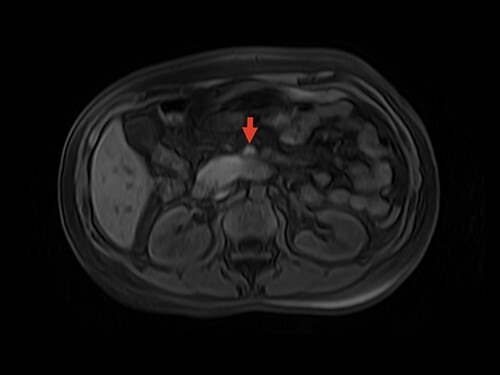
MRI showing superior mesenteric vein thrombus. Arrow: superior mesenteric vein with thrombus inside the vein lumen.

**Figure 2:**
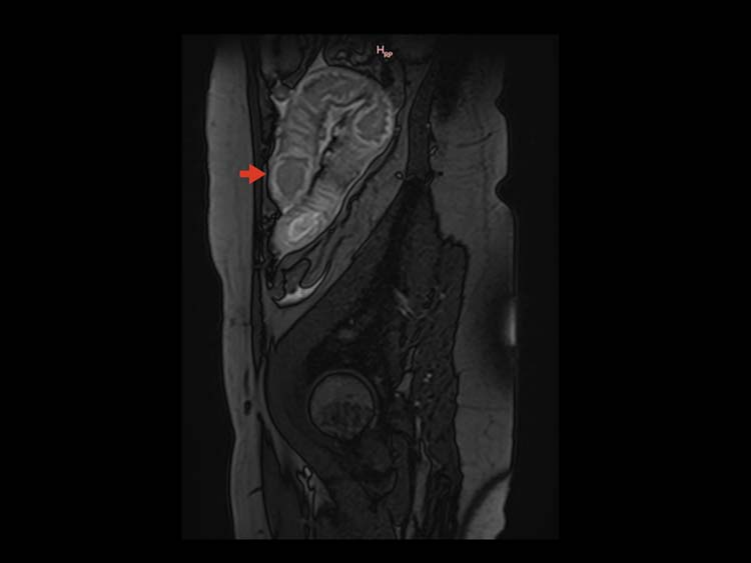
Sagittal section on MRI showing parietal thickening of the intestinal wall. Arrow: thickened intestinal wall.

**Figure 3:**
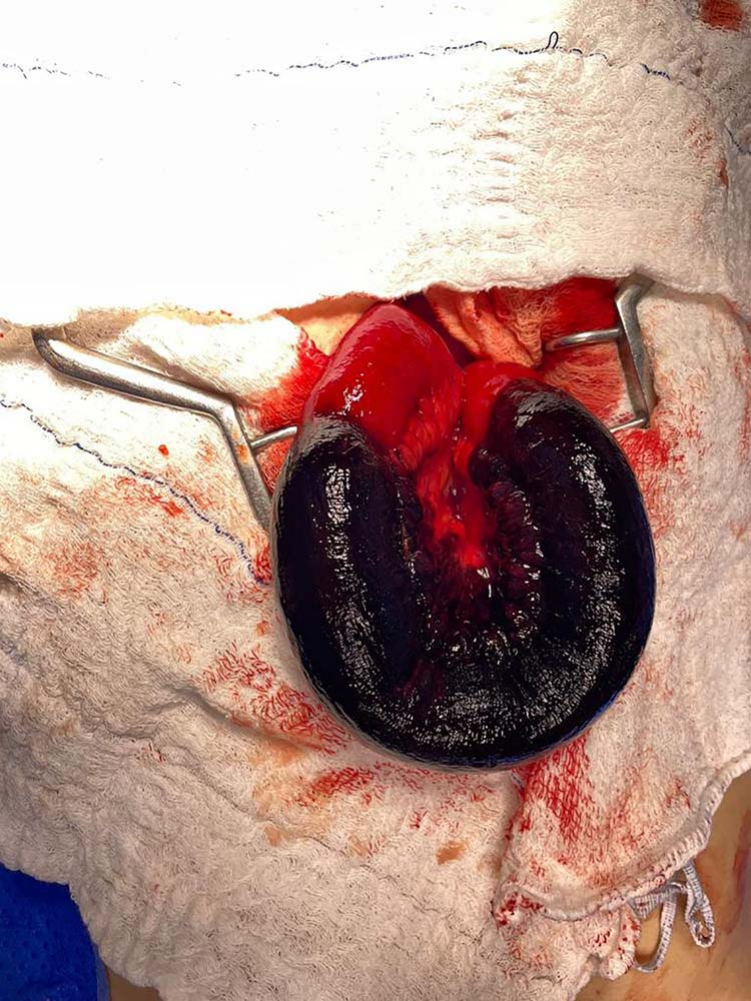
Laparotomy with exposure of the ischemic intestinal loop; resection was performed followed by end-to-end manual anastomosis.

Magnetic resonance image (MRI) was performed. MVT and venous ischemia of a small bowel loop were identified (Figs 1 and 2). She underwent a diagnostic laparoscopy that showed hemorrhagic content in the pelvis and omental plastron surrounding an ischemic bowel loop. The procedure was converted and enterectomy was performed (Fig. 3).

**Figure 4:**
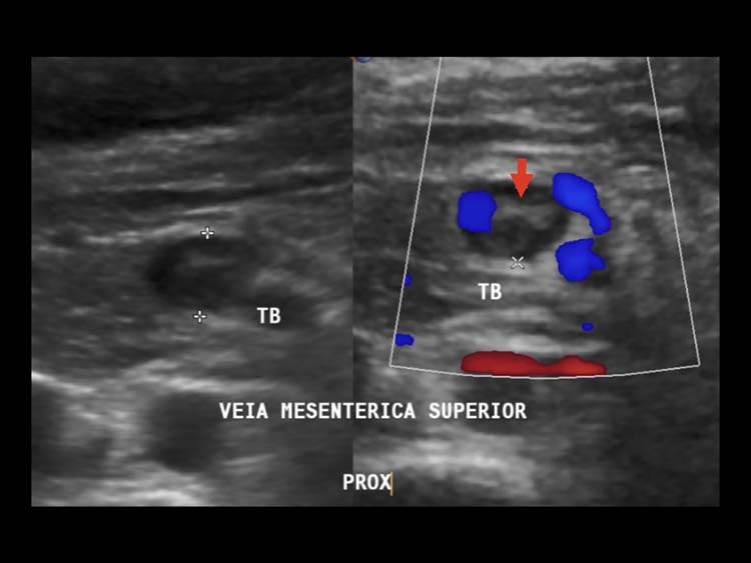
Doppler ultrasound showing superior MVT. Arrow: mesenteric vein thrombus.

Doppler ultrasonography performed on the first and fifth PODs identified superior and inferior MVT (Fig. 4). Anticoagulation with enoxaparin during the remaining pregnancy and puerperium were prescribed.

The recovery was uneventful. The intraabdominal Penrose drain was withdrawn and the patient received hospital discharge on the sixth POD. She was reevaluated in the 14th POD and had no complaints. The thrombophilia investigation is scheduled to be performed after puerperium in order to avoid false-positives results as consequence of physiological changes of pregnancy.

## DISCUSSION

It is well known that physiological changes occur during pregnancy which contribute to a state of hypercoagulability [2–4]. As a consequence of the paucity of cases, the pathophysiology of MVT in these patients is poorly understood. It is likely that cases of IMV described as idiopathic are attributable to previously undiagnosed thrombophilia [3, 4]. However, nothing in the patient’s story above matches thrombophilia.

Venous mesenteric ischemia (VMI) occurs less frequently than arterial ischemia, representing 5–15% of all acute mesenteric ischemia cases [4]. It is a result of blood stasis due to the thrombus, leading to edema and intestinal ischemia, resulting in necrosis and perforation of the organ [3]. Predisposing risk factors include hereditary or acquired thrombophilia, malignancy and cirrhosis [3]. In pregnant women, Factors VII, VIII and fibrinogen are increased, while the activity of the fibrinolytic mechanism is reduced, resulting in 4–5-fold increased risk for thrombosis compared with nonpregnant women [4]. In addition, some factors such as protein S decrease to 40–60% of normal level in order to manage effective bleeding control during delivery [4].

Guan *et al*. [5] evaluated this condition in 15 cases of MVT during pregnancy, and 6 (40%) resulted in fetal death and 1 (6.6%) resulted in maternal death. These results reinforce the risks of this rare condition.

Clinical signs and symptoms include abdominal pain and distention, nausea and vomiting, while common laboratory findings are leukocytosis and elevated CRP [3, 9]. Thus, this working up of abdominal pain with such nonspecific results makes MVT a challenging diagnosis [3–5]. Imaging should be considered at minimal clinical suspicion, such as disproportionate pain or abdominal tenderness, due to possibly severe consequences for the mother and fetus [9]. MRI is the most important diagnostic tool for pregnant women with abdominal pain since ionizing radiation should be avoided [1, 4, 5].

The decision regarding surgical versus endovascular management relies on the patient’s clinical status. In patients without peritonitis, endovascular therapy can be considered as the first-line approach [10]. Emergency surgery is the treatment of choice in the presence of peritonitis when hemodynamically unstable state or septic shock is present [5, 10]. Resection of the necrotic bowel loop and hemodynamic stabilization are the priority, followed by a vascular approach if necessary. For patients with peritonitis who are hemodynamically stable, revascularization should be carried out first and then the bowel viability should be assessed [10].

In the absence of surgical indication, MVT can be managed with supportive measures, anticoagulation and broad-spectrum antibiotics. A 6-month therapy is enough for patients with reversible causes of thrombosis, as opposed to lifelong therapy for those with hypercoagulable disorders or for whom an etiology was not established (idiopathic MVT) [5, 9, 10].

## CONCLUSION

In conclusion, we report a rare case of appendicitis followed by VMT with VMI in a 33-year-old pregnant woman who underwent exploratory laparotomy. A high index of clinical suspicion is necessary for an accurate diagnosis and prompt treatment of this life-threatening condition. Although rare, physicians should be aware of the hypercoagulable state during pregnancy and its natural risk.

## CONFLICT OF INTEREST STATEMENT

The authors declare that they have no conflict of interest and that the ethical principles were followed.

## FUNDING

This study did not receive any specific grant from funding agencies in the public, commercial or non-profit sectors.

## ETHICS APPROVAL

This study complies with institutional/national ethical standards. There is no need for evaluation by National Research Ethics Commission to short report.

## References

[1] Zachariah SK, Fenn M, Jacob K, Arthungal SA, Zachariah SA. Management of acute abdomen in pregnancy: current perspectives. Int J Womens HealthJ Womens Health 2019;11:119–34. 10.2147/IJWH.S151501 PMC6371947 30804686

[2] Chen YY, Wu SM, Kosik RO, Hsieh YC, Wu TI, Chan WP. Acute mesenteric vein thrombosis in a pregnant patient at 10 weeks gestation: a case report. Diagnostics (Basel) 2021;11:1348. 34441283 10.3390/diagnostics11081348 PMC8391634

[3] Feldman ZM, Wang LJ, Chou EL, Latz CA, Sumpio BJ, Eagleton MJ, et al. Venous mesenteric ischemia carries high procedural burden and elevated mortality in patients with severe presentation. J Vasc Surg Venous Lymphat Disord 2021;9:1479–87. 33741519 10.1016/j.jvsv.2021.03.002

[4] Giannos A, Stavrou S, Goumalatsos N, Fragkoulidis G, Chra E, Argiropoulos D, et al. Mesenteric cysts and mesenteric venous thrombosis leading to intestinal necrosis in pregnancy managed with laparotomy: a case report and review of the literature. J Med Case Rep 2017;11:184. 28683785 10.1186/s13256-017-1320-5 PMC5501070

[5] Guan X, Huang L, Li L. Acute mesenteric venous thrombosis in a pregnant woman at 35 weeks of gestation: a case report and review of the literature. BMC Pregnancy Childbirth 2018;18:487. 30537943 10.1186/s12884-018-2126-1 PMC6290498

[6] Bakti N, Hussain A, El-Hasani S. A rare complication of acute appendicitis: superior mesenteric vein thrombosis. Int J Surg Case RepInt J Surg Case Rep 2011;2:250–2. 10.1016/j.ijscr.2011.08.003 PMC3215223 22096743

[7] Germain MA, Soukhni N, Bouzard D. Thrombose veineuse mésentérique compliquant une appendicite aiguë [Mesenteric venous thrombosis complicating acute appendicitis]. Ann ChirAnn Chir 2002;127:381–4. 10.1016/s0003-3944(02)00776-9 12094422

[8] Echtibi SS, Bashir MO, Ahmed MU, Branicki FJ, Abu-Zidan FM. Superior mesenteric vein thrombosis complicating appendicular masses. Saudi Med JSaudi Med J 2003;24:1016–8. 12973491

[9] Lin H, Lin CC, Huang WT. Idiopathic superior mesenteric vein thrombosis resulting in small bowel ischemia in a pregnant woman. Case Rep Obstet GynecolCase Rep Obstet Gynecol 2011;2011:687250. 10.1155/2011/687250 PMC3335606 22567515

[10] Tabriziani H, Ahmad A, Narasimha D, Bergamaschi R, Frishman WH. A nonsurgical approach to mesenteric vascular disease [published correction appears in Cardiol Rev. 2018 Jul/Aug; 26(4):218. Narasimha, Deepika [added]]. Cardiol RevCardiol Rev 2018;26:99–106. 10.1097/CRD.0000000000000180 29419563

